# Oxidative Stress, Kinase Activation, and Inflammatory Pathways Involved in Effects on Smooth Muscle Cells During Pulmonary Artery Hypertension Under Hypobaric Hypoxia Exposure

**DOI:** 10.3389/fphys.2021.690341

**Published:** 2021-08-09

**Authors:** Patricia Siques, Eduardo Pena, Julio Brito, Samia El Alam

**Affiliations:** Institute of Health Studies, Arturo Prat University, Iquique, Chile

**Keywords:** hypobaric hypoxia, pulmonary artery smooth muscle cell, oxidative stress, kinases, inflammation, high altitude

## Abstract

High-altitude exposure results in hypobaric hypoxia, which affects organisms by activating several mechanisms at the physiological, cellular, and molecular levels and triggering the development of several pathologies. One such pathology is high-altitude pulmonary hypertension (HAPH), which is initiated through hypoxic pulmonary vasoconstriction to distribute blood to more adequately ventilated areas of the lungs. Importantly, all layers of the pulmonary artery (adventitia, smooth muscle, and endothelium) contribute to or are involved in the development of HAPH. However, the principal action sites of HAPH are pulmonary artery smooth muscle cells (PASMCs), which interact with several extracellular and intracellular molecules and participate in mechanisms leading to proliferation, apoptosis, and fibrosis. This review summarizes the alterations in molecular pathways related to oxidative stress, inflammation, kinase activation, and other processes that occur in PASMCs during pulmonary hypertension under hypobaric hypoxia and proposes updates to pharmacological treatments to mitigate the pathological changes in PASMCs under such conditions. In general, PASMCs exposed to hypobaric hypoxia undergo oxidative stress mediated by Nox4, inflammation mediated by increases in interleukin-6 levels and inflammatory cell infiltration, and activation of the protein kinase ERK1/2, which lead to the proliferation of PASMCs and contribute to the development of hypobaric hypoxia-induced pulmonary hypertension.

## Introduction

At high altitude – 3,000 m above sea level – the partial pressure of oxygen (PaO_2_) is decreased, creating a condition termed hypobaric hypoxia; this condition is characterized by reduced bioavailability of oxygen at the organism, tissue, and cell levels, which clearly alters organism homeostasis (Chavala, 2018; Bilo et al., 2019). Currently, more than 140 million people worldwide are working and living under the condition of hypobaric hypoxia (Moore et al., 1998; West, 2012), and the consequences associated with this exposure constitute a substantial public health burden (Herrera et al., 2015a; Brito et al., 2020).

High-altitude exposure results in different pathologies, such as acute mountain sickness, chronic mountain sickness (or Monge’s disease), acute cerebral edema (ACE), and high-altitude pulmonary edema (HAPE); however, other important pathology associated with high-altitude exposure is high-altitude pulmonary hypertension (HAPH) (León-Velarde et al., 2005; Robinson et al., 2017). HAPH corresponds to classification 3.6 for general pulmonary hypertension (Simonneau et al., 2013). The cut-off point for all types of pulmonary hypertension is a mean pulmonary arterial pressure (mPAP) ≥ 25 mm Hg (Galiè et al., 2016); however, the cut-off point for HAPH is considered to be an mPAP ≥ 30 mm Hg according to a consensus reached in 2005 (León-Velarde et al., 2005). This condition is observed in subjects who are chronically exposed to high-altitude and is due to hypoxic pulmonary vasoconstriction and subsequent pulmonary artery remodeling (Sommer et al., 2008; Neupane and Swenson, 2017).

Hypoxic pulmonary vasoconstriction is the first response to hypoxia – and certainly to hypobaric hypoxia – due to the decrease in alveolar oxygen pressure (Moudgil et al., 2005; Siques et al., 2018). This intrinsic vasoconstrictor mechanism is activated to distribute oxygen to more adequately ventilated areas of the lung to improve oxygen delivery (Von Euler and Liljestrand, 1946). However, when alveolar hypoxia is maintained, the hypoxic pulmonary vasoconstriction mechanism is exacerbated and can trigger vascular remodeling (León-Velarde et al., 2010; Tan et al., 2017) and, subsequently, pulmonary hypertension, or HAPH (Penaloza et al., 1964; Xu and Jing, 2009; León-Velarde et al., 2010). As mentioned above, pulmonary hypertension is a disease characterized by pulmonary vascular remodeling leading to a progressive increase in pulmonary vascular resistance; however, when this pathology is maintained, the afterload on the right ventricle is increased, and right ventricular hypertrophy occurs, ultimately resulting in right heart failure and death (Mikhael et al., 2019).

Importantly, all layers of the pulmonary artery (the adventitia, smooth muscle, and endothelial cell layers) and the surrounding cells (mast cells, fibroblasts, and macrophages) contribute to or are involved in the development of HAPH (Vodenicharov, 2012; Siques et al., 2018). However, some of the principal action sites of HAPH are pulmonary artery smooth muscle cells (PASMCs), which interact with several extracellular and intracellular molecules and participate in diverse mechanisms – based on the evolution and degree of hypoxia – that lead to proliferation, apoptosis, and fibrosis (Udjus et al., 2019). The aims of this review are to discuss the molecular alterations related to oxidative stress, inflammation, and protein kinase activation that occur under hypobaric hypoxia-induced pulmonary hypertension, particularly in PASMCs, and to identify current and potential molecular targeted pharmacological treatments.

## PASMCs and Hypobaric Hypoxia

The structure of smooth muscle tissue is different from that of other muscle tissue subtypes, and the spindle shape of mononucleated smooth muscle cells (SMCs) permits close contact among cells in vessel walls (Vodenicharov, 2012). The thickness of the medial layer, which is composed mainly of SMCs, greatly determines pulmonary artery resistance and pressure (Siques et al., 2018). Under hypoxic conditions, the muscularization and contractibility of these vessels are enhanced (Meyrick and Reid, 1979), constituting a key feature of pulmonary vascular remodeling and subsequent hypobaric hypoxia-induced pulmonary hypertension (Pak et al., 2007). This effect has been observed both in chronic hypobaric hypoxia (Penaloza and Arias-Stella, 2007) and, albeit to a lesser extent, in intermittent hypobaric hypoxia (Brito et al., 2015). However, this effect in acute hypobaric hypoxia is controversial (Pak et al., 2007), since some studies showed PASMC proliferation under acute hypoxia, while others did not, as demonstrated in a meta-analysis (Pak et al., 2007). Therefore, more studies are necessary to clarify the effect of acute hypobaric hypoxia exposure on SMCs.

As mentioned above, vascular remodeling due to pulmonary artery vasoconstriction is a common characteristic of pulmonary hypertension under hypobaric hypoxia, during which important changes occur in the muscular vessel medial layer that result principally from accumulation of vascular SMCs. Thus, hypobaric hypoxia plays an important role in triggering the excessive proliferation of vascular SMCs and contributing to vascular remodeling (Tan et al., 2017). The processes mentioned are related to oxidative stress, kinase protein, and inflammation in SMCs under hypobaric hypoxia and are discussed below.

## Oxidative Stress

Under physiological conditions, the presence of controlled concentrations of reactive oxygen species (ROS) molecules in the organism is necessary to modulate several pathways involved in maintaining homeostasis and cellular differentiation (Li et al., 2016). ROS are oxidative and unstable molecules derived from oxygen. The most well-known ROS molecules are superoxide anions (O_2_^.–^), hydroxyl radicals (^.^OH), peroxyl radicals (ROO^.^), and alkoxy radicals (RO^.^). Additionally, some non-radicals are either oxidizing or easily converted into oxidative species; these non-radicals include hypochlorous acid (HOCl), ozone (O_3_), singlet oxygen (^1^O_2_), hydrogen peroxide (H_2_O_2_), and peroxynitrite (ONOO^–^) (Li et al., 2016). However, when the level of ROS increases due to environmental or chemical stimuli and is not correspondingly reduced by endogenous antioxidant systems, such as those involving superoxide dismutase (SOD), catalase (CAT), glutathione peroxidase/reductase (GSH-PX/RX), and peroxiredoxin/thioredoxin (Herrera et al., 2015b; Peoples et al., 2019), oxidative stress is triggered. This oxidative stress results in damage to DNA, proteins, and lipids as well as mitochondrial permeability transition pore (MPTP) activation, mitochondrial dysfunction, and cell death (Richardson and Schadt, 2014; Farías et al., 2016, 2017).

Despite the apparent paradox – that O_2_ bioavailability is decreased under hypoxic conditions – there is evidence showing that increased ROS levels trigger oxidative stress in hypobaric hypoxia (Klemm et al., 2011; Lüneburg et al., 2016; Siques et al., 2018). Notably, a study on the lungs of rats exposed to hypobaric hypoxia showed that the H_2_O_2_ concentration increased and that SOD and GSH-PX activity decreased, producing oxidative stress (Xu et al., 2016). Interestingly, hypoxia and consequent pulmonary hypertension are related to oxidative stress (Bowers et al., 2004; Guzy and Schumacker, 2006; Demarco et al., 2010). This is supported by the results of a human study showing that ROS levels are two- to threefold higher in patients with pulmonary hypertension than in control subjects (Wong et al., 2013) and an animal study showing that the levels of a plasmatic oxidative biomarker (malondialdehyde) are elevated in rats with pulmonary hypertension induced by intermittent or chronic hypobaric hypoxia exposure (Lüneburg et al., 2016). Moreover, Xu et al. (2016) demonstrated that ROS and H_2_O_2_ levels are elevated in PASMCs cultured under hypoxic conditions and that the increases in the levels of these molecules are related to PASMC proliferation, contributing to the development of pulmonary hypertension.

### NADPH Oxidase

Regarding the sources of ROS, studies have proposed that mitochondria and nicotinamide adenine dinucleotide phosphate (NADPH) oxidase are the major ROS producers in the cardiovascular system (Daiber et al., 2017; Fukai and Ushio-Fukai, 2020). Additionally, it has been demonstrated the involvement of mitochondria-derived ROS in the development of normobaric hypoxia-induced pulmonary hypertension (Adesina et al., 2015; Snow et al., 2020). Although, previous studies have claimed that the principal source of ROS in the cardiovascular system is the NADPH oxidase complex (Moudgil et al., 2005), and NADPH oxidase-induced oxidative stress may play an important role in pulmonary artery hypertension induced by hypoxia. In addition, studies on PASMCs under normobaric hypoxia in both humans (Ismail et al., 2009) and animals (Mittal et al., 2007) have shown that the NADPH oxidase complex (specifically the Nox4 isoform) is overexpressed, contributing to PASMC proliferation leading to pulmonary hypertension. Moreover, NADPH oxidase expression and subsequent oxidative stress are increased in the lungs of rat exposed to hypobaric hypoxia, effects that are also related to PASMC proliferation through depletion of the transcription factor CREB, triggering pulmonary hypertension (Klemm et al., 2011).

Although there have not been many studies showing the specific expression and activity of the Nox4 isoform related to PASMC proliferation and pulmonary hypertension under hypobaric hypoxia, however, it is important to mention that Nox4 is the principal isoform expressed in PASMCs of patients with pulmonary hypertension (Sturrock et al., 2006). Moreover, a recent study by He et al. (2020) showed that inhibition of oxidative stress induced by hypoxia exposure reduces PASMC proliferation by decreasing hypoxia inducible factor-1α (HIF-1α) levels and Nox4 expression. In addition, a study in mice exposed to both intermittent and chronic hypobaric hypoxia showed that Nox4 expression in the lungs increased and was higher after chronic than intermittent exposure (Lüneburg et al., 2016). Therefore, based on the above evidence, Nox4 could be related to the PASMC proliferation and pulmonary hypertension induced by hypobaric hypoxia exposure.

Regarding Nox4 pathways, studies have showed that transforming growth factor-β (TGF-β) promotes PASMC proliferation through Nox4 expression (Sturrock et al., 2006; Zhou et al., 2013), and Nox4-mediated production of H_2_O_2_ depends on TGF-β levels (Martin-Garrido et al., 2011). Notably, hypoxia also increases TGF-β levels in PASMCs, contributing to proliferation (Zhang et al., 2018). Zhang et al. (2018) also determined that rats exposed to chronic hypobaric hypoxia exhibited increased TGF-β levels and phosphorylation of smad3 (a key downstream molecule in the TGF-β signaling pathway) in pulmonary arteries, contributing to pulmonary hypertension and remodeling. Importantly, that study also showed that primary pulmonary adventitial fibroblasts and macrophages contributed to the TGF-β secretion under this hypoxic condition; these mechanisms could thus play an important role in PASMC proliferation and pulmonary artery remodeling (Zhang et al., 2018).

As mentioned before, Nox4 could play an important role in the PASMC proliferation under hypobaric hypoxia-induced pulmonary hypertension. However, it is important to highlight that Nox2 isoform mediating vascular remodeling is also demonstrated in both normobaric (Liu et al., 2005) and hypobaric (Norton et al., 2013) hypoxia-induced pulmonary hypertension. Although – to our knowledge – we did not find studies particularly in PASMCs proliferation under this latter condition, which results interesting to consider in future studies.

Another molecule that could contribute to oxidative stress is nitric oxide (NO), which is an important endogenous vasodilator that act directly in SMCs and is altered under hypobaric hypoxia. One study has revealed that NO bioavailability is decreased in the pulmonary arteries of rats with hypobaric hypoxia-induced pulmonary hypertension (Siques et al., 2014). This phenomenon could be explained by the reaction between NO and NADPH oxidase-produced O_2_^.–^, which produces ONOO^–^, contributing to oxidative stress (Barman et al., 2014; Siques et al., 2014; Lüneburg et al., 2016). Therefore, based on the above evidence, NADPH oxidase could play an important role in the oxidative stress that occurs in PASMCs under hypobaric hypoxia.

### Pathways Related to Oxidative Stress

Reactive oxygen species are also involved in regulation of cell signaling through the mitogen-activated protein kinase (MAPK) pathway, which is associated with a variety of cellular effects, including proliferation, survival, death, and transformation (Son et al., 2013). One study on rats exposed to hypobaric hypoxia showed that the constitutive MAPK termed apoptosis signal-regulating kinase 1 (ASK1), which is known to be critical in the remodeling processes of pulmonary artery hypertension, participates in PASMC proliferation through activation of p38 MAPK and endothelin-1 (ET-1) in response to oxidative stress (Wilson et al., 2020).

#### ET-1

Endothelin-1 markedly affects SMCs of the vascular wall *via* its potent vasoconstrictor activity and is related to hypertrophic, mitogenic, and antiapoptotic effects on vascular SMCs (Wu et al., 2007). ET-1 is produced primarily by endothelial cells but can also be secreted by epithelial cells, macrophages, and fibroblasts. Importantly, studies have shown that SMCs can also produce ET-1 (Yanagisawa et al., 1988; Miller et al., 1993; Kosacka and Brzecka, 2021). The activity of ET-1 is increased in hypoxia; this increase is related to the level of HIF-1, since ET-1 contains a HIF-1 binding site at base pair 118 (Yamashita et al., 2001). Additionally, a study has demonstrated that ET-1 may play autocrine and paracrine roles (in SMCs and endothelial cells) in the remodeling of PASMCs during the development of pulmonary hypertension under hypobaric hypoxia (Nakanishi et al., 1999).

As mentioned above, ET-1 may be associated with oxidative stress, since a study in fetal PASMCs showed that increases in the levels of this protein are related to production of large amounts of ROS and that the use of antioxidants or NADPH oxidase inhibitors induces apoptosis of fetal PASMCs (Wedgwood et al., 2001). The above findings are supported by a study showing that hypoxia-induced ET-1 promotes SMC proliferation *via* NADPH oxidase activation, contributing to pulmonary hypertension induced by hypoxia exposure (Wu et al., 2007). Moreover, another study determined that circulating ET-1 levels were significantly higher in HAPH patients than in healthy high-altitude inhabitants (Kojonazarov et al., 2012), which is supported by the aforementioned study by Nakanishi et al. (1999).

Endothelin-1 has been found to be related to upregulation of nuclear factor of activated T cells isoform c3 (NFATc3) in the pulmonary arteries of mice under chronic hypobaric hypoxia (De Frutos et al., 2011). NFATc3 is a Ca^2+^-dependent transcription factor that has been implicated in the PASMC proliferative response and pulmonary hypertension under chronic hypobaric hypoxia in adult mice (De Frutos et al., 2007; Bierer et al., 2011). The role of transcription factors in this process will be discussed later.

#### Peroxisome Proliferator-Activated Receptor

Other molecules related to oxidative stress in PASMCs are the peroxisome proliferator-activated receptors (PPARs), which regulate several genes involved in cell metabolism, growth, and differentiation (Lefebvre et al., 2006). PPARγ is expressed in several cells, including pulmonary endothelial cells and PASMCs, where it plays an important role in normal pulmonary vascular function (Green et al., 2011, 2012). A study on human PASMCs exposed to hypoxia determined that PPARγ downregulation is related to increases in Nox4-mediated H_2_O_2_ production, ERK1/2 levels, and nuclear factor κB (NF-κB) levels (Lu et al., 2013). However, PPARγ activation attenuates signaling pathways associated with SMC proliferation and remodeling (Nisbet et al., 2010). Moreover, loss of PPARγ expression is associated with the development of pulmonary hypertension induced by hypoxia; this finding is supported by a study showing that the expression of PPARγ is decreased in the lungs of rodents with pulmonary hypertension induced by chronic hypoxia exposure (Nisbet et al., 2010). Therefore, alterations in PPARγ under hypoxia are related to kinase activation, oxidative stress, and inflammation, which could contribute to SMCs proliferation under hypobaric hypoxia.

As previously mentioned, hypobaric hypoxia-induced oxidative stress is also related to protein kinase activation and contributes to the initiation of PASMC proliferation and subsequent pulmonary hypertension. These pathways are discussed in the next section.

## Protein Kinase Activity

### JAK2

An important kinase protein related to pulmonary hypertension is mammalian Janus kinase (JAK). This kinase undergoes autophosphorylation at tyrosine residues, which generates binding sites for phosphorylated signal transducer and activator of transcription (STAT). The JAK family contains four evolutionarily conserved members, JAK1, JAK2, JAK3, and tyrosine kinase 2 (TYK2). It has been demonstrated that mRNA expression of JAK1, JAK2, and JAK3 is increased in PASMCs after hypoxia exposure. In addition, inhibition of JAK2 blocks STAT3 expression in PASMCs under hypoxia (Wang et al., 2005). Interestingly, the levels of tyrosine 705-phosphorylated STAT3 (PY-STAT3) are increased in the lungs of rats with pulmonary hypertension induced by hypobaric hypoxia (Huang et al., 2019).

JAK2 is involved in the regulation of various processes relevant to cell survival, proliferation, activation, and differentiation (O’Shea and Plenge, 2012; Thomas et al., 2015). A recent study demonstrated that the JAK2/STAT3 pathway is involved in the proliferation of PASMCs under hypoxic conditions; additionally, mice with SMC-specific *Jak2* knockout show reduced right ventricular systolic pressure and right ventricular hypertrophy under hypoxic conditions, implying improved function of pulmonary blood vessels (Zhang et al., 2020). Moreover, the same study indicated that the JAK2/STAT3 pathway induces pulmonary artery remodeling by enhancing cyclin A2 expression in PASMCs.

### ERK

Notably, some molecular channels are also related to protein kinase activation during PASMC proliferation under hypobaric hypoxia, as indicated by a study showing that transient receptor potential melastatin 7 (TRPM7), which acts as a magnesium channel in mammalian cells, is downregulated in PASMCs of patients with pulmonary hypertension and in rats with hypobaric hypoxia-induced pulmonary hypertension. In particular, inhibition of TRPM7 increased the proliferation and apoptosis resistance of PASMCs *in vitro* through activation of the MEK/ERK pathway (Xing et al., 2019). The role of ERK activation in PASMC proliferation could be supported by the finding that ERK activity is increased in rats with hypobaric hypoxia exposure-induced pulmonary hypertension by a metabolite derived from the lipoxygenase pathway [12(S)-hydroxyeicosatetraenoic acid (12(S)-HETE)], which is related to PASMC proliferation (Preston et al., 2006).

ERK1/2 also mediates the activation of protein kinase C (PKC) and G-protein coupled receptors, and this mechanism induces SMC proliferation under hypoxia (Dempsey et al., 1996; Lannér et al., 2005). Importantly, oxidative agents such as H_2_O_2_ and superoxide result in PKC activation independent of the classical PKC cofactor diacylglycerol; indeed, a study showed that PKC activation induced by H_2_O_2_ results in the activation of the L-type Ca^2+^ channel in SMCs, highlighting that inhibition of PKC activity inactivates this channel (Perez-Vizcaino et al., 2010). Therefore, we suggest that hypoxia exposure can alter oxidative status and kinase activation, leading to impairment of Ca^2+^ homeostasis and contractibility in PASMCs.

### SphK1

Sphingosine kinase-1 (SphK1) is an important lipid kinase that is also related to PASMC proliferation. Its product sphingosine-1-phosphate (S1P) induces PASMC proliferation through activation of early growth response protein-1 (Erg-1), a product of platelet-derived growth factor (PDGF)-induced ERK activation (Sysol et al., 2016). This pathway is also activated during hypoxia-induced PASMC proliferation and pulmonary hypertension, and could be regulated by microRNA (miR-1) (Sysol et al., 2018). Interestingly, a recent study has shown that increases in S1P result in dephosphorylation of yes-associated protein (YAP), a transcriptional regulator, which induces an increase in miR-130a/b contributing to PASMC proliferation and pulmonary hypertension (Shi et al., 2021). The activity of the transcription factor YAP will be discussed later.

## Inflammation

Inflammatory processes are present in various types of pulmonary hypertension in both humans and animals, contributing to pulmonary vascular remodeling (Savale et al., 2009; Tuder, 2017). It is important to mention the role of inflammatory cells, since in mice exposed to hypoxia, monocytes migrate to the lung perivascular space and differentiate into interstitial macrophages (Florentin et al., 2018). These cells contribute to the development and progression of acute or chronic inflammatory responses through the secretion of inflammatory cytokines [TNF-α, IL-1β, and interleukin-6 (IL-6)] in the pathogenesis of inflammatory lung disease (Lee et al., 2021). Interestingly, macrophages are involved in PASMC proliferation, and studies have demonstrated that the numbers of macrophages in perivascular cells increase under hypobaric hypoxia (Horita et al., 2013; Yu Y. R. et al., 2013).

### MIF

Additionally, both macrophages and endothelial cells can produce a proinflammatory cytokine termed macrophage migration inhibitory factor (MIF). Under stress conditions (such as hypoxia), this molecule inhibits the random migration of macrophages and stimulates TNF-α, IL-1β, IL-6, and IL-8 production (Bloom and Bennett, 1966). MIF has been found to be related to proliferation of SMCs (Calandra et al., 1994; Chen et al., 2004; Onodera et al., 2004), and a study on rats under hypobaric hypoxia showed that an increase in MIF induces proliferation of PASMCs through activation of ERK1/2 and JNK proteins, contributing to the pulmonary hypertension (Zhang et al., 2012).

### PTEN

The macrophage accumulation and IL-6 production are regulated by PTEN, since a study on rats exposed to hypobaric hypoxia has shown that selective inhibition of PTEN in PASMCs increases their proliferation and impairs pulmonary hypertension; highlighting that depletion of PTEN exacerbated macrophage accumulation and IL-6 production (Horita et al., 2013).

PTEN is a phosphatase enzyme that suppresses multiple signaling networks involved in cell proliferation, survival, and inflammation (Huang et al., 2019). It also is a specific regulator and protector against pathological vascular SMC remodeling, which is inactivated in the lungs (Huang et al., 2019) and PASMCs in human pulmonary hypertension (Horita et al., 2013). However, a study on the lungs of rats exposed to hypobaric hypoxia found that PTEN expression was decreased but that the membrane localization of its regulator caveolin-1 (cav-1) was unchanged, which could contribute to hypoxia-induced pulmonary hypertension (Huang et al., 2019). This latter finding needs further validation in PASMCs proliferation under hypobaric hypoxia.

### IL-6

The level of IL-6 was demonstrated to be increased in mouse lungs and cultured PASMCs after exposure to normobaric hypoxia, elucidating that IL-6 stimulates human PASMC migration (Savale et al., 2009). In addition, mice with IL-6 deficiency exposed to hypoxia showed reductions in pulmonary hypertension, vascular remodeling, and macrophage recruitment in the lung (Savale et al., 2009). Importantly, increases in IL-6 have also been showed to occur in the lungs of mice exposed to chronic hypobaric hypoxia (Maston et al., 2018).

Interleukin-6 acts through two mechanisms in organisms to exert its biological effects. For example, activation of the membrane-bound IL-6 receptor (IL6R) is related to the classical signaling pathway that controls homeostatic processes and immunological outcomes, such as host defense against bacterial infections and activation of anti-inflammatory and regenerative epithelial pathways (Scheller et al., 2011). On the other hand, IL-6 and its soluble receptor sIL-6R form a complex of the IL-6 trans-signaling pathway that is related to pathological responses such as tissue fibrosis, inflammatory arthritis, and cardiovascular disease (Hunter and Jones, 2015). Moreover, the binding of IL-6 to either the membrane-bound IL6R or sIL-6R triggers the oligomerization of the ubiquitous transmembrane protein gp130, resulting in activation of the JAK/STAT signal transduction pathway (Campbell et al., 2014). Notably, there is evidence showing that gp130 expression is increased in the lungs of rats exposed to hypobaric hypoxia (Huang et al., 2019).

A recent study showed the importance of IL6R and its potential prosurvival effects in pulmonary hypertension under hypoxic conditions (Tamura et al., 2018). Tamura et al. (2018) exposed transgenic mice (deficient in IL6R in SMCs) to chronic normobaric hypoxia and found that they exhibited decreased activation of STAT3 and expression of antiapoptotic proteins, such as MCL-1 and BCL2, in the lungs. Moreover, the mice showed decreased collagen deposition, inflammatory cell infiltration, and pulmonary hypertension (Tamura et al., 2018).

Interestingly, inhibition of the pathway involving the IL-6/sIL-6R complex reduces the pulmonary artery remodeling and pulmonary hypertension that occur under hypobaric hypoxia, highlighting that activation of the IL-6/sIL-6R complex enhances PASMC migration (but not proliferation) and that this migration process requires STAT3 activation and increases in [Ca^2+^]_i_ (Maston et al., 2018). These results indicate that IL-6, IL6R, and sIL-6R could play an important role in hypoxia-induced PASMC proliferation and pulmonary hypertension.

### Caspase-1

Another interesting protein is the inflammasome effector caspase-1, which mediates inflammation through activation of the proinflammatory cytokines IL-18 and IL-1β (Udjus et al., 2019). A study showed that caspase-1 deficiency reduces pulmonary hypertension in mice under normobaric hypoxia, mainly by reducing PASMC proliferation. In addition, caspase-1 activation likely induces PASMC proliferation *via* the IL-18/IL-1β and IL-6/STAT3 signaling pathways. Therefore, caspase-1 could be another therapeutic target for hypoxia-induced pulmonary hypertension (Udjus et al., 2019).

### MKL1

The transcriptional regulator megakaryocytic leukemia 1 (MKL1) is also an important modulator of SMCs under hypobaric hypoxia (Yuan et al., 2014). MKL1 inhibition attenuates pulmonary hypertension in rats exposed to chronic hypobaric hypoxia (Yuan et al., 2014). It also decreases the expression of inflammatory cytokines (TNF-α and IL-6) and chemokines responsible for recruitment of inflammatory cells, such as C–C motif chemokine ligand 2 (CCL2) and CCL5, resulting in decreased aggregation of cell types including macrophages, leukocytes, and T lymphocytes in the lungs (Yuan et al., 2014).

In addition, a study on calves has indicated that hypobaric hypoxia-induced pulmonary hypertension is associated with increased production of extracellular matrix proteins, specifically collagen type I, by SMCs (Crouch et al., 1989). On the other hand, a study based on this finding by Yuan et al. (2014) showed that depletion of MKL1 prevents the increased synthesis of endogenous collagen type I mRNA in vascular SMCs under hypoxic conditions (Yuan et al., 2014). Therefore, MKL1 synthesized in SMCs likely participates in pulmonary hypertension induced by hypobaric hypoxia through fibrogenesis and inflammation.

## Transcription Factors

As mentioned previously, YAP is a transcriptional regulator that, together with WW domain-containing transcription regulator 1 (WWTR1 or TAZ), contributes to PASMC proliferation and subsequent pulmonary hypertension under hypoxic conditions through inhibition of cyclooxygenase 2 (COX2) activity (Dieffenbach et al., 2017). Additionally, a recent study has shown that YAP induces the activation of forkhead box M1 (FOXM1), a transcription factor that contributes to PASMC proliferation and pulmonary hypertension (Zhang et al., 2021).

### FOX

The forkhead box (FOX) proteins are a superfamily of transcriptional regulators that mediate numerous functions during development and adulthood (Lam et al., 2013). FOXM1 is a transcription factor that plays a crucial role in the maintenance and differentiation of epithelial cells lining the airways, especially during the embryonic stage, during which it is essential in the formation and proliferation of pulmonary vessels (Raoof and Daoud, 2019). In mice with hypoxia-induced pulmonary artery hypertension, FOXM1 mRNA and protein levels are increased in the lungs; moreover, constitutive SMC-specific knockdown of FOXM1 inhibits hypoxia-induced pulmonary hypertension and prevents the proliferation of PASMCs, although it does not inhibit PASMC migration (Dai et al., 2018). Importantly, FOXM1 in PASMCs is regulated by HIF-1α under normoxic conditions but stimulated by HIF-2α under hypoxic conditions (Xia et al., 2009). Therefore, FOXM1 can be modulated by HIF-1α and HIF-2α, but its response to oxygen (according to its expression level) and its distribution in tissues differ (Raghavan et al., 2012). Hypoxia upregulates FOXM1 expression in SMCs by increasing the release of growth factors and inflammatory cytokines from endothelial cells, contributing to pulmonary artery hypertension (Raoof and Daoud, 2019).

Another important transcription factor that regulates the cell cycle is forkhead box protein O1 (FOXO1), which has been demonstrated to play a role opposite that of FOXM1 (Sengupta et al., 2013). FOXO1 expression is also decreased in the lungs of humans with another type of pulmonary artery hypertension. Supporting the role of FOXO1 under hypoxia, it has been demonstrated that inhibition of FOXO1 significantly increases PASMC proliferation (Savai et al., 2014).

There is a relationship among FOXM1, FOXO1, and the cell cycle regulator polo-like kinase 1 (PLK1) (Wilson et al., 2019). The latter has been shown to be an important upregulator of FOXM1 expression, contributing to PASMC proliferation in subjects with pulmonary artery hypertension (Wilson et al., 2019). Inhibiting the actions of either PLK1 or FOXM1 in human PASMCs increases the expression of the cyclin-dependent kinase inhibitor 1B (p27^kip1^) protein, which inhibits the cell cycle progression of PASMCs. In addition, specific inhibition of FOXO1 increases the expression of FOXM1 (Wilson et al., 2019).

More studies are necessary to assess the roles of FOX proteins in pulmonary hypertension under hypobaric hypoxia. However, a recent study has shown that the levels of forkhead box C1 (FOXC1) are increased in the pulmonary arteries of mice exposed to hypobaric hypoxia and in PASMCs cultured under hypoxic conditions (Yang et al., 2020). In addition, the viability and migration ability of PASMCs transfected with a FOXC1 overexpression plasmid have been found to be increased under hypoxic conditions. Moreover, the circular RNA mmu_circ_0000790 regulates the expression of FOXC1 by binding to miR-374c, establishing an important regulatory axis of vascular remodeling and pulmonary hypertension under hypobaric hypoxia (Yang et al., 2020). Although these new molecules and pathways were originally described in the context of other types of hypertension, they have been demonstrated to also play a role in hypoxic pulmonary hypertension.

### Wnt/β-Catenin

Another pathway that is important to mention is the Wnt/β-catenin pathway. β-Catenin is a transcription factor involved in PASMC proliferation, and its expression can be inhibited by Wnt5a (in a non-canonical Wnt pathway) in hypoxia-induced pulmonary hypertension (Yu X. M. et al., 2013). Wnt5a levels are decreased under hypoxic conditions (Yu X. M. et al., 2013). Therefore, β-catenin activity is predominant under hypoxia, as supported by a recent study showing that the expression of β-catenin is increased in the lungs of rats exposed to hypobaric hypoxia (Huang et al., 2019). On the other hand, induction of Wnt5a overexpression in recombinant mice significantly inhibits β-catenin expression, human PASMC proliferation, and subsequent hypoxia-induced pulmonary hypertension (Yu X. M. et al., 2013; Jin et al., 2015). Therefore, the Wnt/β-catenin pathway could be important to consider in future studies on hypobaric hypoxia-induced PASMC proliferation and pulmonary hypertension.

### Erg-1

The redox-sensitive transcription factor Erg-1 regulates cell proliferation, and the levels of this transcription factor are increased in newborn calves with pulmonary artery hypertension and remodeling induced by chronic hypobaric hypoxia. The increases in Erg-1 are related to protein kinase activation and oxidative stress, since oxidant/antioxidant imbalance due to chronic hypobaric hypoxia-induced loss of extracellular SOD activity in the pulmonary artery results in Erg-1 activation and contributes to PASMC proliferation through the ERK1/2 pathway (Hartney et al., 2011).

**FIGURE 1:**
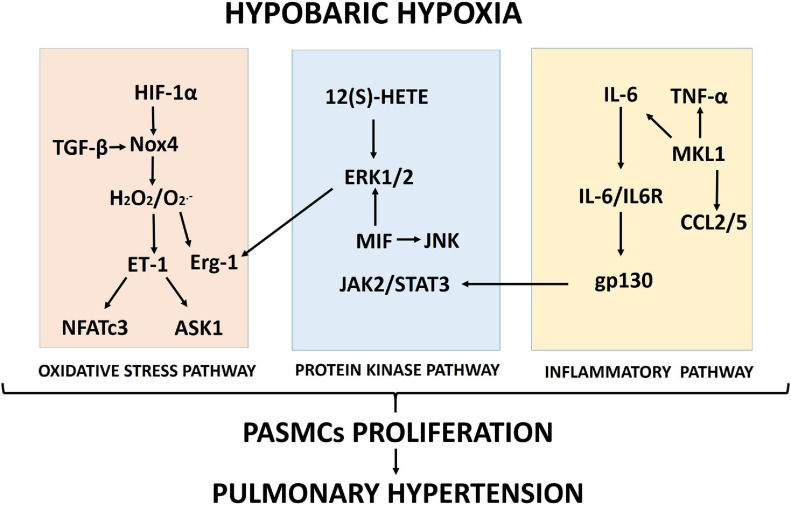
Proposed scheme summarizing the molecular pathways (oxidative stress, protein kinase and inflammation) involved in pulmonary artery smooth muscle cell (PASMC) proliferation induced by hypobaric hypoxia exposure.

**FIGURE 2:**
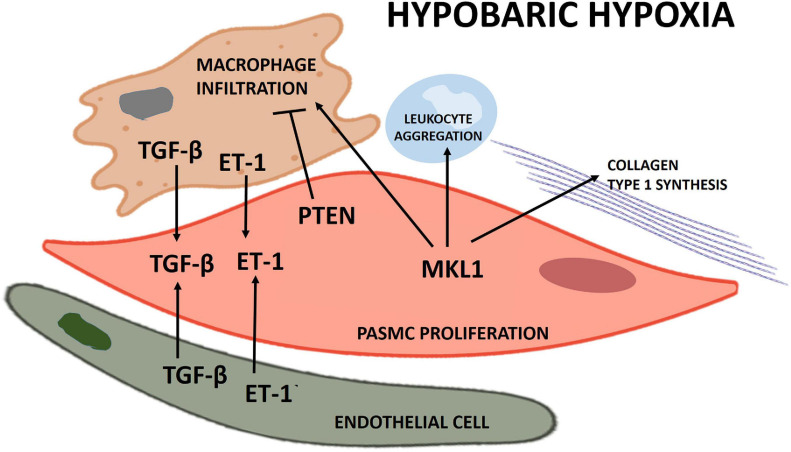
Scheme of the principal cellular interactions involved in pulmonary artery smooth muscle cell (PASMC) proliferation under hypobaric hypoxia.

Proposed schematics of PASMC proliferation under hypobaric hypoxia summarizing the molecular pathways and their cellular interactions discussed in this review are depicted in Figures 1, 2, respectively.

## Pharmacological Interventions to Decrease PASMCs Proliferation Under Hypoxic Conditions

Additionally, we review the current interventions for PASMCs proliferation under hypoxic conditions.

One interesting study has shown that treatment with histone deacetylase inhibitors (HDIs), such as suberoylanilide hydroxamic acid (SAHA) and butyrate (BUR), increases the expression of endothelial NO synthase (eNOS) in vascular SMCs, producing an elevation in NO that induces upregulation of p21 expression and cleavage of caspase 3 (a protein related to cell cycle arrest and apoptosis) and decreases hypobaric hypoxia-induced vascular SMC remodeling (Tan et al., 2017). However, importantly, a significant decrease in mPAP does not occur (Tan et al., 2017).

Resveratrol, a plant-derived polyphenolic compound and a phytoestrogen, has been demonstrated to alleviate hypobaric hypoxia-induced pulmonary hypertension through its anti-inflammatory, antioxidant, and antiproliferative effects (Xu et al., 2016). This compound decreased neutrophil infiltration in the lungs of rats exposed to hypobaric hypoxia and suppressed the expression of inflammatory molecules such as phosphorylated NF-kB, IL-6, IL-1β, and TNF-α. Importantly, resveratrol inhibited the production of ROS in PASMCs (probably by increasing Nrf-2/Trx-1 axis signaling) and suppressed the proliferation of PASMCs. Additionally, the decrease in the proliferation of PASMCs by resveratrol could be explained by the decrease in HIF-1α expression through MAPK/ERK1 and PI3K/AKT inhibition (Xu et al., 2016). In fact, in support of this hypothesis, a recent study by He et al. (2020) showed that resveratrol inhibited hypoxia-induced oxidative stress and the proliferation of rat PASMCs by decreasing HIF-1α levels and Nox4 expression.

Other interesting medicines derived from Tibetan plants have been used to reduce hypoxia-induced pulmonary hypertension. Echinacoside, a phenylethanoid glycoside from the Tibetan herbs *Lagotis brevituba* Maxim and *Cistanche tubulosa*, shows antiproliferative effects in rat PASMCs under normobaric hypoxia (Gai et al., 2014). In addition, echinacoside was administered to rats under hypobaric hypoxia (4,500 m), and it reduced pulmonary artery remodeling and pulmonary hypertension (Gai et al., 2020). Administration of another traditional Tibetan medicine, Tsantan Sumtang, prevented pulmonary hypertension in rats exposed to hypobaric hypoxia (4,500 m), reducing pulmonary vascular cell proliferation probably by suppressing cyclin D1 and cyclin-dependent kinase 4 (CDK4) expression through inhibition of p27^kip1^ degradation (He et al., 2018).

Consistent with the relation between JAK2/STAT3 pathway activation and cell proliferation, administration of tanshinone IIA (Tan), a fat-soluble pharmacologically active ingredient in Danshen, decreases hypoxia-induced PASMC proliferation *via* apoptosis activation through inhibition of the JAK2/STAT3 pathway both *in vivo* (in rats) and *in vitro* (Chen et al., 2014).

Aloperine, an alkaloid isolated from *Sophora alopecuroides* L., possesses a variety of pharmacological activities, such as anti-inflammatory and antitumor activities (Chang et al., 2019). Human PASMCs in which proliferation was induced by stimulation with platelet-derived growth factor-BB (PDGF-BB) were treated with aloperine and exhibited decreased proliferation and increased apoptosis. These effects could be explained by the decreased levels of NF-kB, TNF-α, and cyclin E1 and the increased level of p27^kip1^ (Chang et al., 2019).

**FIGURE 3:**
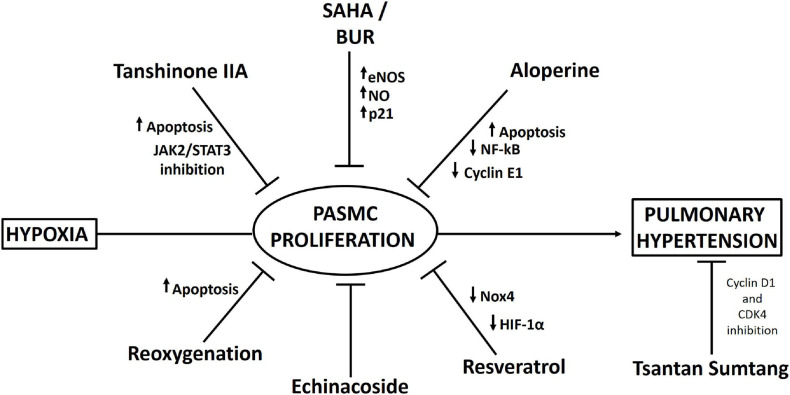
Scheme summarizing pharmacological interventions that have been described to decrease pulmonary artery smooth muscle cell (PASMC) proliferation and pulmonary hypertension under hypobaric hypoxia.

Finally, an interesting study, which requires further validation, showed that reoxygenation results in increased apoptosis in PASMCs, thus reversing hypobaric hypoxia-induced pulmonary artery remodeling, which could be attributed to mitochondrial ROS-mediated mitochondrial dysfunction (Chen et al., 2017). A proposed scheme of the pharmacological approaches is depicted in Figure 3.

## Conclusion

Hypoxia exposure is an important inducer of PASMC proliferation and subsequent remodeling and pulmonary hypertension. Moreover, evidence suggests that hypobaric hypoxia exposure activates several molecular pathways related to oxidative stress (the Nox4 pathway), protein kinases (ERK1/2), and inflammation (IL-6 and inflammatory cell infiltration). These pathways independently or cooperatively lead to further proliferation of PASMCs, contributing to subsequent pulmonary hypertension. In addition, based on the evidence discussed, a summarized array of some different pharmacological approaches has been developed to mitigate this proliferative effect. However, further studies are necessary to validate these treatments in people with PASMCs proliferation and subsequent HAPH induce by hypobaric hypoxia.

## Author Contributions

PS, EP, JB, and SEA contributed to conceiving the review, reviewing the literature, writing and preparing the draft, and reviewing and editing the manuscript. All authors have read and agreed with the submitted version of this manuscript.

## Conflict of Interest

The authors declare that the research was conducted in the absence of any commercial or financial relationships that could be construed as a potential conflict of interest.

## Publisher’s Note

All claims expressed in this article are solely those of the authors and do not necessarily represent those of their affiliated organizations, or those of the publisher, the editors and the reviewers. Any product that may be evaluated in this article, or claim that may be made by its manufacturer, is not guaranteed or endorsed by the publisher.

## Abbreviations

HAPH: high-altitude pulmonary hypertension
PASMCs: pulmonary artery smooth muscle cells
PaO_2_: partial pressure of oxygen
ACE: acute cerebral edema
HAPE: high-altitude pulmonary edema
mPAP: mean pulmonary arterial pressure
SMCs: smooth muscle cells
ET-1: endothelin-1
NO: nitric oxide
HIF-1: hypoxia inducible factor-1
NFATc3: nuclear factor of activated T cells isoform c3
ROS: reactive oxygen species
NADPH: nicotinamide adenine dinucleotide phosphate
TGF-β: transforming growth factor-β
eNOS: endothelial nitric oxide synthase
O_2_.^–^: superoxide
.OH: hydroxyl radical
H_2_O_2_: hydrogen peroxide
CAT: catalase
GSH-PX/RX: glutathione peroxidase/reductase
HDIs: histone deacetylase inhibitors
FOX: forkhead box
FOXM1: forkhead box M1
FOXO1: forkhead box O1
PLK1: polo-like kinase 1
FOXC1: forkhead box C1
Erg-1: early growth response-1
SOD: superoxide dismutase
MPTP: mitochondrial permeability transition pore
MAPK: mitogen-activated protein kinase
ASK1: apoptosis signal-regulating kinase 1
PPARs: peroxisome proliferator-activated receptors
YAP: yes-associated protein
NF-κB: nuclear factor κB
JAK: Janus kinase
STAT: signal transducer and activator of transcription
TRPM7: transient receptor potential melastatin 7
PKC: protein kinase C
IL-6: interleukin-6
IL6R: membrane-bound interleukin-6 receptor
sIL-6R: soluble interleukin-6 receptor
MKL1: megakaryocytic leukemia 1
TNF-α: tumor necrosis factor alpha
SAHA: suberoylanilide hydroxamic acid
BUR: butyrate
JNK: c-Jun N-terminal kinase.
